# The World Health Organization Prequalification Program and Clinical Pharmacology in 2030

**DOI:** 10.1002/cpt.1680

**Published:** 2019-11-26

**Authors:** Terrence F. Blaschke, Murray Lumpkin, Daniel Hartman

**Affiliations:** ^1^ Stanford University Stanford California USA; ^2^ Integrated Development Bill & Melinda Gates Foundation Seattle Washington USA

**Access to affordable, high‐quality medicines, vaccines, and medical devices is critical to patients in resource‐limited settings. For the past 23 years, the World Health Organization Prequalification Program (WHOPQ) has provided procurers with a rigorous assessment of the quality of these products. As the number of important products has soared, the resources of the WHOPQ program are being strained. Advances in the field of clinical pharmacology could help meet the increased demand on this important program.**

With support from multiple international organizations, the WHOPQ program was established for vaccines in 1996 and for medicines in 2001.^1^ Programs for *in vitro* diagnostics (IVDs) and vector control products have also been implemented. All involve an assessment of product performance, an assurance of manufacturing to international quality standards, and a determination of product suitability in low‐ and middle‐income countries (LMICs). The initial program goal was to provide a service to United Nations procurement agencies to facilitate their knowledge of manufacturers whose versions of certain medical products met international standards of quality, safety, and efficacy. These procurement agencies and often countries themselves used WHOPQ listing as a resource when selecting product vendors to assure that their funds were buying quality‐assured product versions. Equally important, WHOPQ listings are now relied upon by many countries to facilitate rapid local registration of medical products (**Supplementary Figure** S1).

Any manufacturer is permitted to submit their product dossier to the WHOPQ program for assessment if that product is one in which the relevant WHO medical program has expressed interest for use in LMIC health programs. Due to the focus of the procurement agencies involved, initially, WHOPQ was limited to medicines for treating HIV/AIDS, tuberculosis, and malaria. In 2006, this was extended to medicines for reproductive health and, in 2008, to products for managing children's diarrhea. Vaccines for most childhood diseases have been prioritized for WHOPQ. **Figure** 1 shows the numbers of products that have been prequalified as of January 2019.

**Figure 1:**
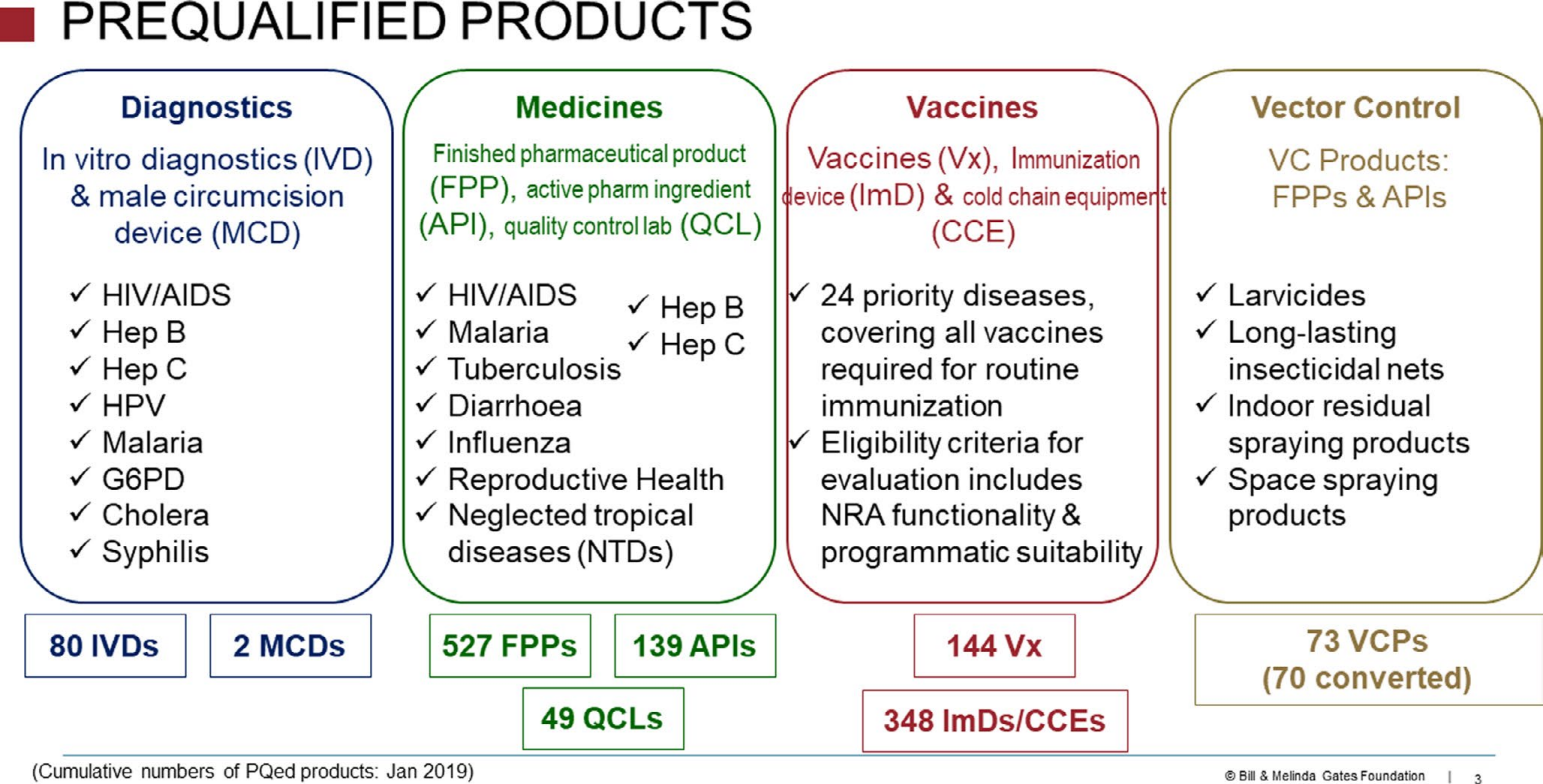
This figure shows the number of products “listed” (i.e., approved) by the World Health Organization Prequalification (WHOPQ) program as of January 2019 across the four PQ product streams. Note the expansion of medicines that are now prequalified for transmissible infectious diseases, but the absence of medicines important for the management of many noncommunicable diseases because, under current WHO policy, they are not eligible for PQ assessment. Reprinted with permission of the Bill and Melinda Gates Foundation.

Two recent articles focusing on the WHOPQ have recommended program changes to help increase access to products in LMICs. Roth *et al*.^2^ encouraged the WHO to expand the WHOPQ to include new classes of medicines, such as biologics (which has been initiated), medical devices beyond male circumcision devices and IVDs (which has been initiated), and other essential medicines. This is consistent with similar calls from the current WHO Director General, Dr. Tedros Adhanom Ghebreyesus, as part of his focus on assuring universal access to quality health care, recognizing that without access to quality assured medicines there is no quality health care.

Ahonkhai *et al*.^3^ investigated the product registration ecosystem timelines in 2012, including WHOPQ when required, intended for LMIC diseases, and proposed a framework to reduce product registration time. The framework stressed: (i) re‐engineering no longer fit‐for‐purpose processes; (ii) focusing on value‐added activities, including minimizing redundancy by increasingly relying on work products (scientific assessments and inspections reports) of trusted regulatory authorities and WHOPQ, and by maximizing the scope of countries participating in the WHOPQ collaborative registration program in which countries rely on prequalification (PQ) program assessments to inform their own national registration decisions within a 90‐day time frame; and (iii) focusing on regional, rather than individual country, registration approaches that encompass harmonizing technical standards and optimizing regulatory processes in concert with international standards (i.e., those of WHO and/or the International Council on Harmonisation). The WHO and many LMIC National Regulatory Authorities (NRAs) have now embraced these recommendations. The result has been the development of systems now in place through which a quality dossier can be evaluated in half the time it required in 2012 (**Supplementary Figure** S2). In June 2019, the WHO released its 5‐year plan to help build effective and efficient regulatory systems.^4^ As shown in **Figure** 2, this plan builds on current successes and responds to further LMIC NRA requests.

**Figure 2:**
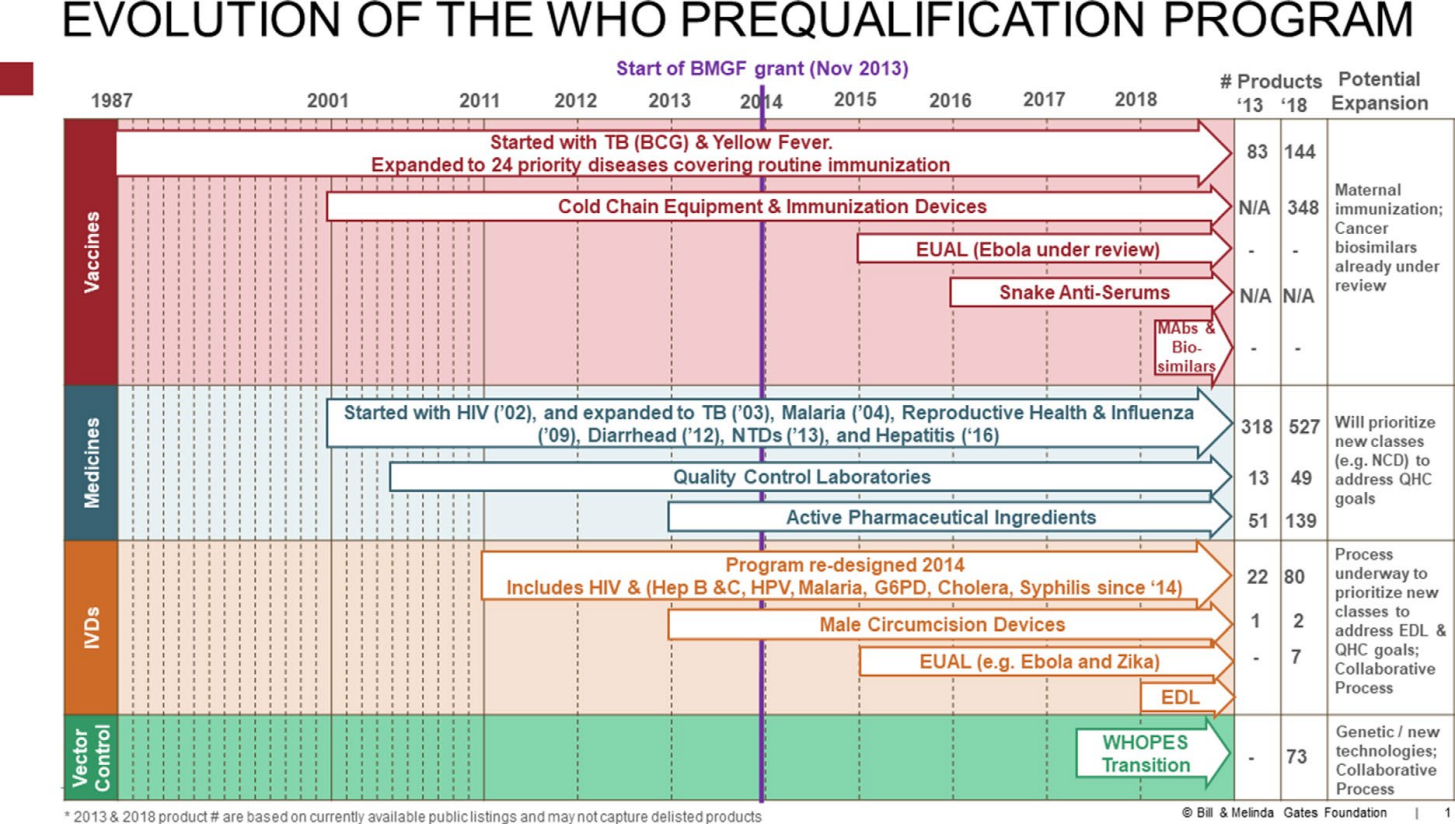
This figure, provided by the Bill and Melinda Gates Foundation, illustrates the evolution of the World Health Organization Prequalification (WHOPQ) programs and its four product streams. As noted in the text, the WHOPQ program was established for vaccines in 1996 and for medicines in 2001. Its remit is to identify manufacturers of products who produce their products in compliance with international standards for product efficacy, safety, and manufacturing quality. Over time, the scope of products eligible for PQ assessment have expanded and broadened. In view of the financial and technical resource constraints that currently limit expansion of the WHOPQ program scope, new advances in the science of clinical pharmacology might prove valuable in the next decade to help facilitate that expansion in a scientifically rigorous, but more efficient manner. EDL, Essential Drug List; EUAL, emergency use assessment and listing; HPV, human papillomavirus; IVD, *in vitro* diagnostics; MAbs, monoclonal antibodies; NCD, noncommunicable diseases; NTD, neural tube defects; QHC, Quality Health Care; TB, tuberculosis; WHO, World Health Organization; WHOPES, the acronym for the old WHO process for assessing the utility of vector control products. Reprinted with permission of the Bill and Melinda Gates Foundation.

Recently, the WHO released its 21st Model List of Essential Medicines (EML), its seventh Model List of Essential Medicines for children, and its second Model List of IVDs. These are published biennially and list those products the WHO believes should be available and affordable everywhere.^5^ Countries use these lists to shape their country‐specific essential medicines and IVD lists. Although the new list added 12 new medicines, including some for cancers, anticoagulation, and chronic inflammatory diseases, the list still favors infections and overlooks products for some conditions with a large global disease and economic burden (e.g., diabetes and mental health). These and other noncommunicable diseases (NCDs) kill 41 million people each year, equivalent to 71% of all deaths globally and 15 million people between the ages of 30 and 69 years die from an NCD. Moreover, 85% of these “premature” deaths occur in LMICs.

The WHO 2030 Agenda for Sustainable Development recognizes NCDs as a major challenge for sustainable development. As a WHOPQ listing is often critical for local registration and procurement of many drugs, the WHO challenges are: (i) how to expand the EMLs to include products for more NCDs and new infectious diseases affecting many in LMICs, (ii) how to expand WHOPQ scope so that more of these EML products are eligible for PQ assessment and so that manufacturers of quality versions of these products can be identified for global and national procurement and use in health care programs in LMICs, and (iii) how to expand the number regulatory agencies of manufacturing countries, which effectively enforce international level quality oversight of local manufacturing, so that WHOPQ can rely on them in performing their assessment and listing, rather than having to perform a full PQ assessment and inspection themselves on most of the products submitted to WHOPQ for assessment. Implementing “WHO's 5‐year plan” plan and achieving its goals will require trained clinical pharmacologists with a broad skill set, including core pharmacology to assess novel and generic products (especially medicines), as well as individuals with pharmacokinetic/pharmacodynamic and physiologically‐based pharmacokinetic modeling skills.

## How can Clinical Pharmacology Help Address some of these Challenges in the Next Decade?

A recent External Assessment Report titled “Assessment of WHO Prequalification and Systems Supporting Activities” included both qualitative and quantitative analyses and highlighted the major areas in which WHOPQ and its systems‐supporting activities have had an impact (direct and indirect) on the global health ecosystem, including saving US $30 to $40 in procurements costs for each $1 spent on the PQ program potentially enabling the purchase of millions of additional doses of medicines. In addition, some areas of improvement were identified that could further enhance WHOPQ's impact.^6^

Acknowledging the financial and technical resource constraints that currently limit WHOPQ scope expansion, new clinical pharmacology advances might prove valuable in the next decade to help address these challenges. Sophisticated quantitative methods and computational modeling have great potential for improving generic drug availability by reducing product development time and cost without sacrificing robust approval data, as these methods have informed more efficient study designs for comparative bioequivalence evaluation.^7^, ^8^

Biowaivers for a larger product spectrum have become available to regulators in Europe and the United States. They eliminate the clinical bioequivalence study requirement for many generic drugs that have excellent absorption characteristics and for some generic drugs that are poorly permeable but have high solubility. Biowaivers reduce the costs/time for generic drug development and approval. Clinical pharmacology can help develop the scientific rationale and database for increasing the number and scope of biowaivers.

Increased capacity for conducting small, focused, well‐planned pharmacokinetic and safety studies in LMICS may be advantageous by providing additional data that help better define product relevance, safety, and efficacy in various populations.

The WHO's quality health care initiative recognizes the impact disease has on children. WHOPQ's ability to assess products for children, the majority of whom live in LMICs, is critical. Most pediatric formulation registration generally follows approval in adults. Clinical pharmacologists' experience in modeling and simulation strategies facilitates better data extrapolation from adults to children using the latest knowledge of the children's physiology and pharmacology. This approach can be used to model product efficacy and toxicity in a similar pediatric indication.^9^ If necessary, relatively small postregistration pediatric studies could be conducted in various regions.

Perhaps the greatest product development and registration impact in LMICs is the evolution of LMIC clinical trial infrastructure to support the growth of local product development and manufacturing. There is a disproportionately low number of LMIC clinical researchers relative to their regions' high burden of disease, aggravated by emigration of up to 70% of scientists for employment elsewhere.^10^ To help address this problem, a novel university‐accredited, immersive fellowship program has been established by a large public‐academic‐private network, involving participation of ~ 140 scientists from 25 countries over a 7‐year period. A recent evaluation revealed strong evidence of knowledge and skills transfer, and beneficial self‐reported impact on fellows' research output and career trajectories. There was a high retention of fellows in their home countries (> 75%) and an enduring professional network among the fellows and their mentors.^10^ Enhancing the clinical trial sites quality to achieve good laboratory practice and good clinical practice accreditation and training more clinical pharmacologists with expertise in trial designs have the potential to significantly reduce the lag time for new chemical entities or a newly formulated generic equivalents to reach LMICs.

Ultimately, as more countries procure their own medicines and vaccines, WHOPQ must be able to assess quality versions of most EML products, as well as other new chemical entities of significant clinical importance, such as new drugs for life‐threatening (e.g., cancer) or life‐altering (e.g., dementias) conditions. In doing so, they must be able to work with local regulatory agencies to help enhance their abilities to regulate confidently when such is appropriate and to regulate effectively within their jurisdictions those aspects of a product's life cycle for which they cannot rely on other agencies (e.g., local pharmacovigilance, local supply chain security and safety, and local and export manufacturing). Likewise, they must work with local procurers to assure their confidence in the oversight of these products, the appropriateness of these products for their populations, and the affordability of the products.

These are significant challenges for the global health community. Clinical pharmacology has much to offer to help meet many of these challenges to help assure access to affordable quality‐assured health care products in LMICs.

## Funding

No funding was received for this study. D.H. and M.M.L. are full‐time employees of the Bill and Melinda Gates Foundation, and preparation of this manuscript was carried out as part of their employment at the Foundation.

## Conflicts of Interest

All authors declared no competing interests for this work.

## Supporting information


**Figure S1.** This graphic, provided by the Bill and Melinda Gates Foundation, illustrates the very dramatic and increasing collaboration between the WHO PQ program and multiple National Regulatory Authorities between 2012 and 2018. As shown in the upper left panel, 36 countries and CARICOM (15 Caribbean nations) are involved in this collaborative process, resulting in 403 rapid drug registrations since 2013 (lower left panel), a vaccine pilot program now underway, and a diagnostics program that began a pilot in 2018. A vector control products collaboration is planned for 2020. The map shows the countries participating, many of which are located in sub‐Saharan Africa. Reprinted with permission of the Bill and Melinda Gates Foundation.
**Figure S2.** This figure is illustrative of the improvements in low‐income country product registration systems timelines between 2013 and 2018. SRA refers to a “stringent” regulatory authority (as defined by WHO) and NRA refers to all other national regulatory authorities. The definition of an SRA has been clarified by the WHO PQ Guidance document released on 15 February 2017. Through greater reliance on the inspections and scientific assessments of trusted authorities, re‐engineering processes that were no longer fit‐for‐purpose and using regional rather than national approaches for joint assessments, registration systems are now in place through which a quality product can proceed in half of the time required in 2012. Reprinted with permission of the Bill and Melinda Gates Foundation.
