# Supplementary material for: The World Health Organization Prequalification Program and Clinical Pharmacology in 2030
Source: Clin Pharmacol Ther. 2019 Nov 26;107(1):68–71. doi: 10.1002/cpt.1680 (PMC7586841; doi:10.1002/cpt.1680)
Supplement: Supplementary file 1 — Figure S1. This graphic, provided by the Bill and Melinda Gates Foundation, illustrates the very dramatic and increasing collaboration between the WHO PQ program and multiple National Regulatory Authorities between 2012 and 2018. As shown in the upper left panel, 36 countries and CARICOM (15 Caribbean nations) are involved in this collaborative process, resulting in 403 rapid drug registrations since 2013 (lower left panel), a vaccine pilot program now underway, and a diagnostics program that began a pilot in 2018. A vector control products collaboration is planned for 2020. The map shows the countries participating, many of which are located in sub‐Saharan Africa. Reprinted with permission of the Bill and Melinda Gates Foundation. Figure S2. This figure is illustrative of the improvements in low‐income country product registration systems timelines between 2013 and 2018. SRA refers to a “stringent” regulatory authority (as defined by WHO) and NRA refers to all other national regulatory authorities. The definition of an SRA has been clarified by the WHO PQ Guidance document released on 15 February 2017. Through greater reliance on the inspections and scientific assessments of trusted authorities, re‐engineering processes that were no longer fit‐for‐purpose and using regional rather than national approaches for joint assessments, registration systems are now in place through which a quality product can proceed in half of the time required in 2012. Reprinted with permission of the Bill and Melinda Gates Foundation. [file CPT-107-68-s001.docx]

**The World Health Organization (WHO) Prequalification Program and Clinical Pharmacology in 2030**

**Supporting Information:**

These two figures Illustrate the increasing breadth of collaboration between the WHO and National Regulatory Authorities worldwide, and the improvement in product registration timelines between 2013 and 2018.

**SUPPLEMENTARY FIGURE 1
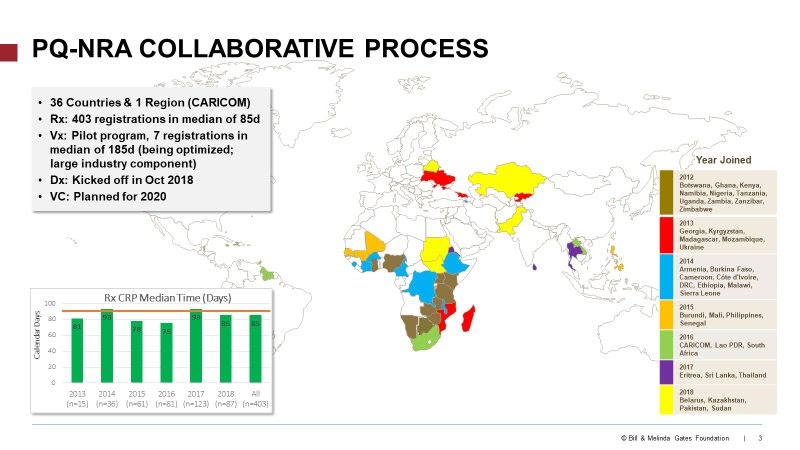
**

**SUPPLEMENTARY FIGURE 1 LEGEND**

This graphic illustrates the very dramatic and increasing collaboration between the WHO PQ program and multiple National Regulatory Authorities (NRA) between 2012 and 2018. As shown in the upper left panel, 36 countries and CARICOM (15 Caribbean nations) are involved in this collaborative process, resulting in 403 rapid drug registrations since 2013 (lower left panel), a vaccine pilot program now underway and a diagnostics program beginning in 2018. A Vector Control (VC) collaboration is planned for 2020. The map shows the regions participating, many of which are located in Sub-Saharan Africa.

**
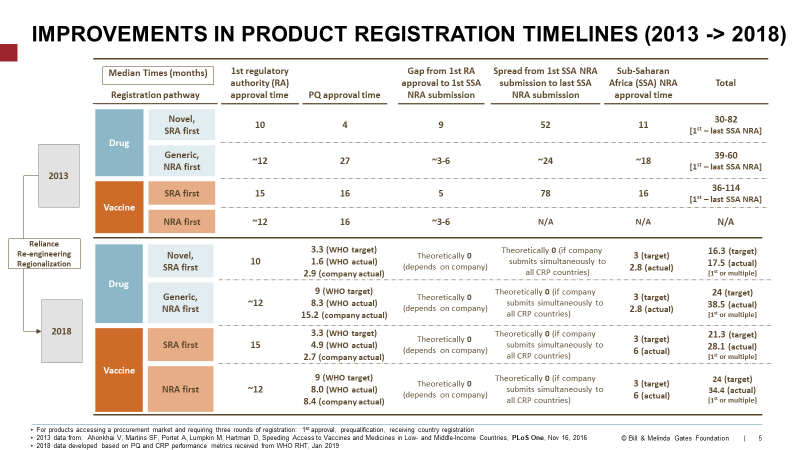
SUPPLEMENTARY FIGURE 2**

**SUPPLEMENTARY FIGURE 2 LEGEND**

This figure is illustrative of the improvements in low-income country product registration systems timelines between 2013 and 2018. SRA refers to a “stringent” regulatory authority (as defined by WHO) and NRA refers to all other national regulatory authorities. The definition of an SRA has been clarified by the WHO PQ Guidance document released on 15 February 2017. Through greater reliance on the inspections and scientific assessments of trusted authorities, re-engineering processes that were no longer fit-for-purpose, and using regional rather than national approaches for joint assessments, registration systems are now in place through which a quality product can proceed in half-the time required in 2012
